# Adopting a Collaborative Strategy to Address the Complexities of Implanting a Subcutaneous Implantable Cardiac Defibrillator for Secondary Prevention in a Patient With Fabry Disease and Motor Neuron Disease

**DOI:** 10.7759/cureus.79379

**Published:** 2025-02-20

**Authors:** Sumantra Kumar De, Elton Luo, Adnan Ahmed, Padmanabhan Shakkottai, Renjith Antony

**Affiliations:** 1 Cardiac Research, Northwick Park Hospital, London, GBR; 2 Cardiology, Hull University Teaching Hospital, Hull, GBR

**Keywords:** fabry disease (fd), motor neuron disease, multidisciplinary team, quality of life in cardiology, quality of life (qol), secondary prevention, subcutaneous icd

## Abstract

A man in his mid-40s, diagnosed with motor neuron disease (MND) and Fabry disease (FD), was sent to cardiology after experiencing a cardiac arrest at home secondary to ventricular fibrillation (VF). Fabry disease is a rare X-linked inherited lysosomal storage disorder caused by deficient alpha-galactosidase A (AGAL-A) activity that leads to an accumulation of globotriaosylceramide (Gb3) in affected tissues, including the heart. Motor neuron disease is an uncommon condition that progressively damages parts of the nervous system. He experienced a positive neurological recovery, enabling the cardiology team to investigate the cause of his arrest. Despite his considerable coexisting health issues and reliance on medical and non-medical interventions, he seemed to enjoy a good quality of life. By working together, the multidisciplinary team, alongside the patient and their family, reached an agreement on the treatment offered. In light of his ongoing medical conditions, the patient underwent a subcutaneous implantable cardioverter defibrillator (S-ICD) implantation to reduce the risk of infection while ensuring he got the relevant protection in the event of further life-threatening ventricular arrhythmia. He had a successful recovery, maintaining his quality of life and independence. Here, we describe the challenges involved in ensuring the best interest decision was taken, which required extensive collaboration from various specialties following a cardiac arrest.

## Introduction

Fabry disease (FD) is a rare X-linked inherited lysosomal storage disorder caused by deficient alpha-galactosidase A (AGAL-A) activity that leads to an accumulation of globotriasylceramide (Gb3) in affected tissues, including the heart. The prevalence of FD among White male populations has been documented to fluctuate significantly, with estimates ranging from approximately one in 17,000 to one in 117,000 [1]. About 1:22,000 to 1:40,000 males have classic FD mutations, while 1:6000 to 1:40,000 females and 1:1000 to 1:3000 males have atypical presentations [1]. Fabry disease can affect both the male and female populations and can be classified into classic or late-onset phenotypes. In classic FD, AGAL-A activity is absent or severely reduced, and disease manifestations have an early onset that can affect multiple organs. Patients with late-onset FD have residual AGAL-A activity. The clinical features are primarily confined to the heart [2]. A particular study consisting of nearly 1,450 FD patients showed nearly ^3^/_4_ of deaths were due to cardiac diseases, while 62% were attributed to sudden cardiac deaths [3].

Motor neuron disease (MND) is an uncommon condition that progressively damages parts of the nervous system. The male population is affected almost twice as often as the female population; however, this can vary [4]. Myocardial involvement in MND is an uncommon feature. There are, however, suggestions that cardiomyopathy may occur in patients with MND [5]. The exact pathogenic mechanism responsible for the increased incidence of cardiomyopathy in MND patients remains unclear. It could be hypothesized that hyperlipidemia may play a role in the development of cardiomyopathy in these individuals [5]. Muscle biopsies of two such patients exhibited secondary myopathic changes along with neurogenic damage [5].

Treatment options for chronic illnesses such as the aforementioned conditions have improved in recent years with advancements in contemporary medicine. This means that increasingly, most modern medical specialties (including cardiology) that look after complex patients with multiple comorbidities will need to adopt a multidisciplinary approach to deliver the best medical care. Here, we describe a challenging clinical scenario involving a gentleman suffering from both multisystem FD and MND requiring extensive collaboration from various specialties following a cardiac arrest. Particularly, this case demonstrates how a multidisciplinary team navigated through the complex decision-making required for the patient’s secondary prevention through an implantable cardioverter defibrillator (ICD). Both MND and FD are also an extremely rare combination in a patient. This novelty of our patient has already been described by the patient’s neurology team in an earlier case report and he remains the only such case in the literature, to the best of our knowledge [6].

## Case presentation

**Figure 1:**
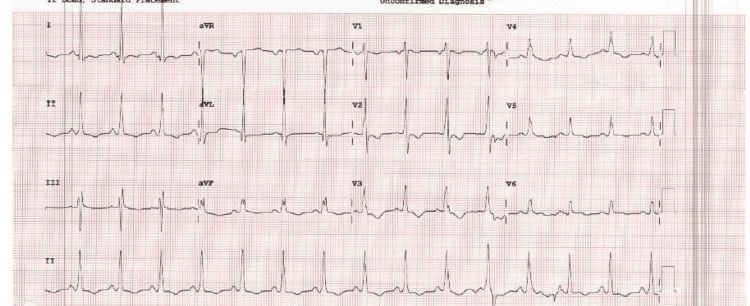
A 12-lead electrocardiogram (ECG) showing sinus rhythm, normal axis, normal PR and QTc interval, with tall R waves in V2 and inverted T waves in V2-V3 suggesting some evidence of right ventricular hypertrophy with strain

A male patient in his mid-40s who was a known case of MND (amyotrophic lateral sclerosis (ALS) variant) and FD with c.299G>A, p.R100K mutation, with cardio-renal involvement for the last decade, was found unresponsive by his carers. He received immediate bystander cardiopulmonary resuscitation (CPR) followed by two external direct current defibrillations for ventricular fibrillation (VF). He then received further CPR for 30 minutes before the return of spontaneous circulation (ROSC). The post-ROSC electrocardiogram (ECG) showed sinus rhythm, normal axis, normal PR, and QTc interval, with tall R waves in V2 and inverted T waves in V2-V3 suggesting some evidence of right ventricular hypertrophy with strain (Figure 1). He received tracheal intubation and was mechanically ventilated for a few days; however, inotropic or circulatory support was not required. The patient had episodes of myoclonus suggestive of focal seizures; however, an electroencephalogram (EEG) demonstrated no definite epileptiform activity, and no clinical events were captured during the recording. Fortunately, he made a satisfactory neurological recovery, leading to successful extubation and de-escalation from critical care. 

Prior to this admission, he was on three times a week hemodialysis via a left internal jugular tunneled central venous catheter. Additionally, he had a port-a-cath on his right subclavian vein for long-term venous access. He had a permanent tracheostomy and received home ventilation for 12 hours during night-time. Our patient also suffered from peripheral arterial disease and previously had angioplasty to his posterior tibial and superficial femoral arteries. He also had a long-term suprapubic catheter in place. He was on Fabrazyme (enzyme replacement therapy for FD). Despite significant comorbidities, our patient was able to communicate using Eyegaze technology (Eyegaze Inc., Fairfax, VA) [7], which would track his eyeball movements. He used a wheelchair for mobility independently up until a year ago. He lived at home with his wife and received 24-hour care from a team of nurses and carers. Importantly, with the help of his carers, our patient led a good quality of life, regularly attending his children’s social events, visiting local shops, and being actively involved in family and finance matters.

Post-ROSC ECG and serial 12-lead ECGs did not show any new changes to his previous ones and no evidence of channelopathies (Figure 1).

Laboratory blood tests revealed borderline low potassium levels with a baseline biochemistry panel (Table 1). His infection markers were mildly raised, for which he was treated with intravenous antibiotics. A possible explanation was he could have aspirated during his cardiac arrest. His troponin levels were elevated secondary to his cardiac arrest and ventricular arrhythmia.

**Table 1:** The patient's laboratory findings during presentation

Test	Results	Normal range	Unit
Haemoglobin	95	135 - 175	g/L
White cell count	24.9	4 – 11	X10^9^/L
Platelets	261	150 – 400	X10^9^/L
Neutrophils	21.7	2-7.7	10^9 /L
Sodium	136	135 – 144	mmol/L
Bicarbonate	21	24 – 32	mmol/L
Potassium	3.3	3.5 – 5.3	mmol/L
Urea	17	3.0 – 7.6	mmol/L
Creatinine	123	65 – 114	µmol/L
Calcium adjusted level	2.31	2.2 – 2.6	mmol/L
Magnesium	0.9	0.7-1.0	mmol/L
Troponin	2772	14	ng/L
C-reactive protein	96	0 – 8	mg/L
Alanine aminotransferase	92	5 – 45	IU/L
Albumin	27	36 – 48	g/L
Alkaline phosphatase	182	30 – 125	IU/L
Bilirubin	6	<21	umol/L
Total protein	65	65 – 82	g/L

**Figure 2:**
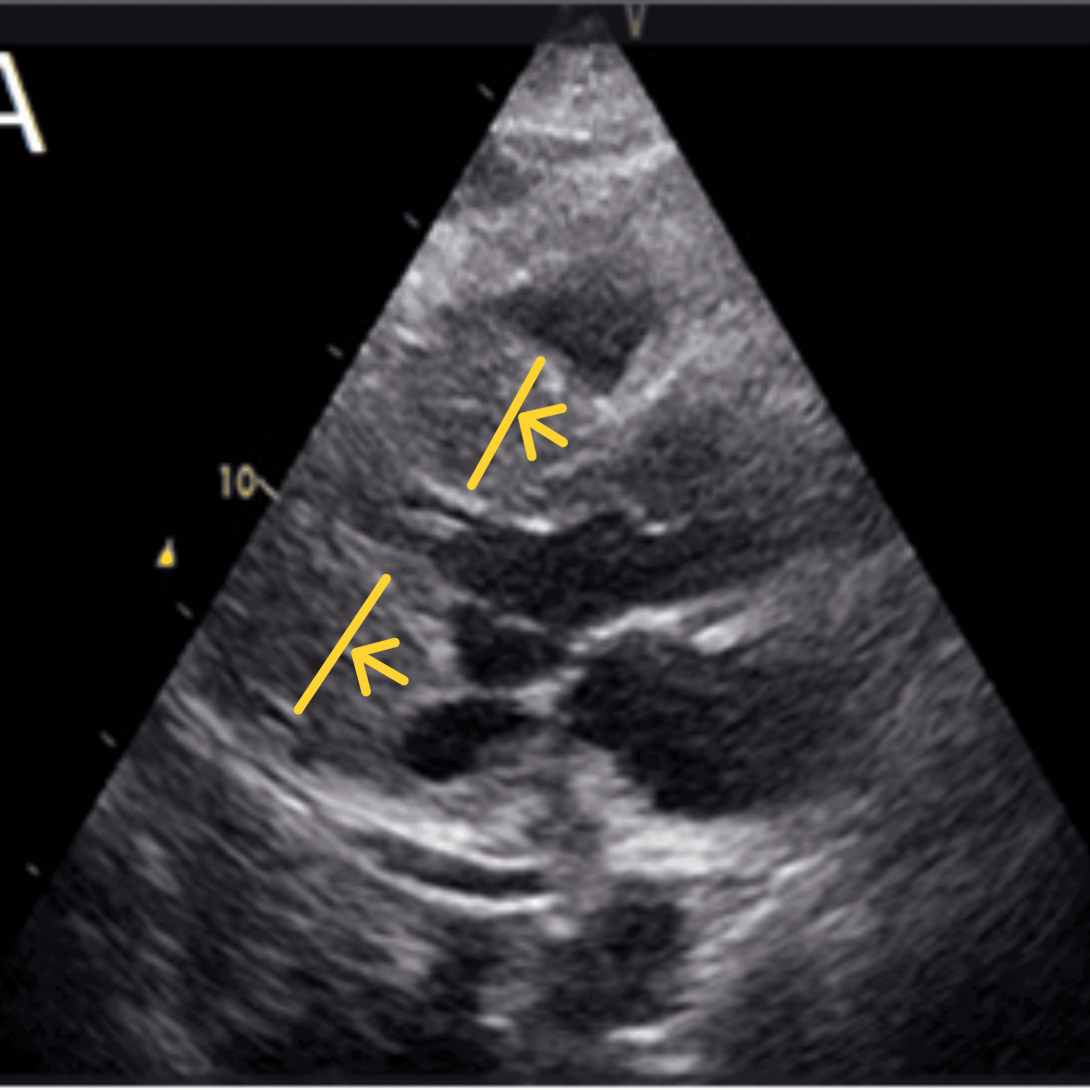
Significant concentric left ventricular hypertrophy is shown by yellow arrows on the parasternal long-axis views on transthoracic echocardiogram.

**Figure 3:**
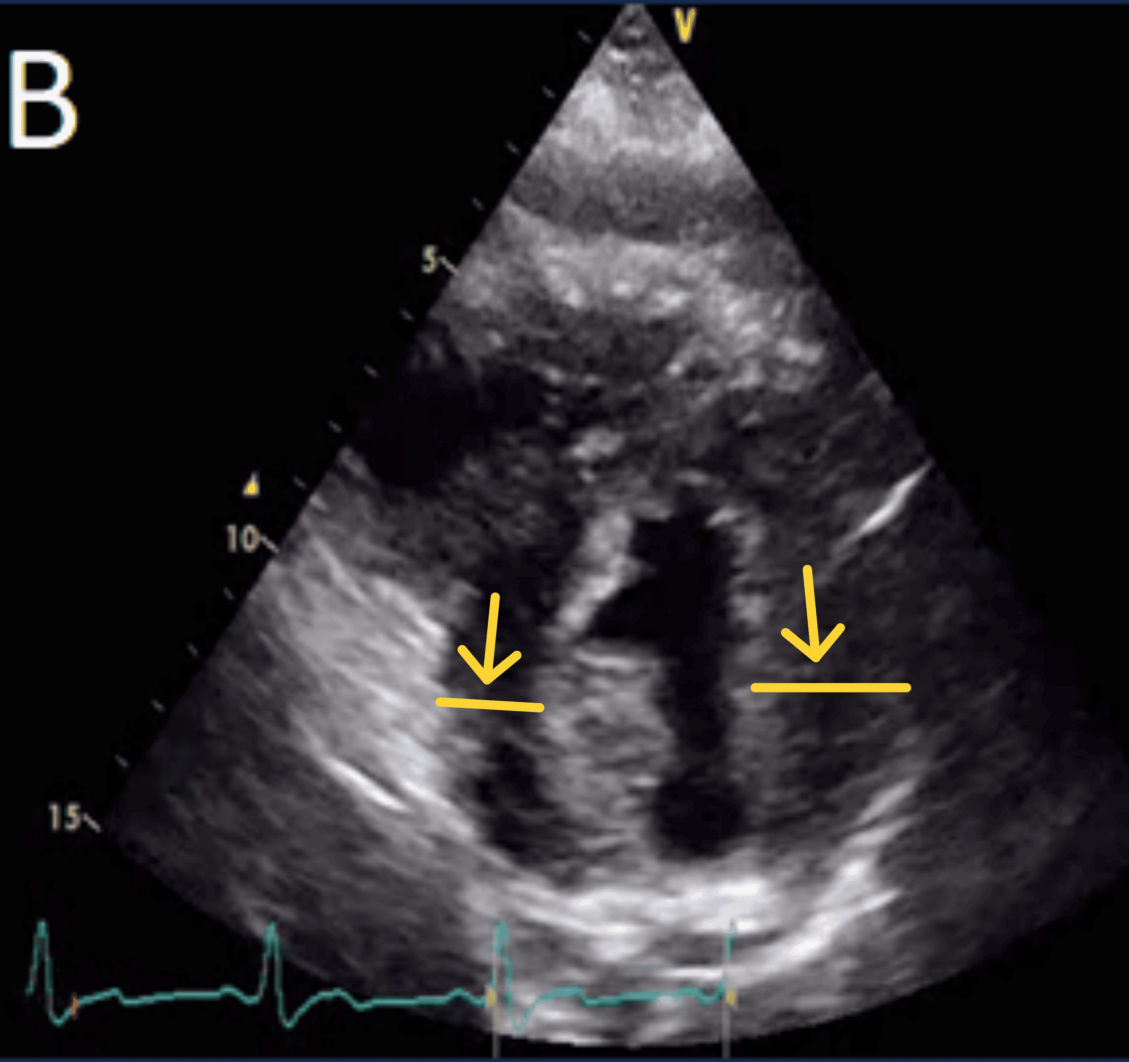
Significant concentric left ventricular hypertrophy is shown by yellow arrows on the parasternal short-axis views on transthoracic echocardiogram.

A 2D transthoracic echocardiogram (TTE) performed showed a non-dilated left ventricle with significant concentric hypertrophy and almost complete obliteration of the left ventricular cavity (Figures 2, 3). The left ventricular systolic function was noted to be borderline low. There was no significant valvular or pericardial disease.

**Figure 4:**
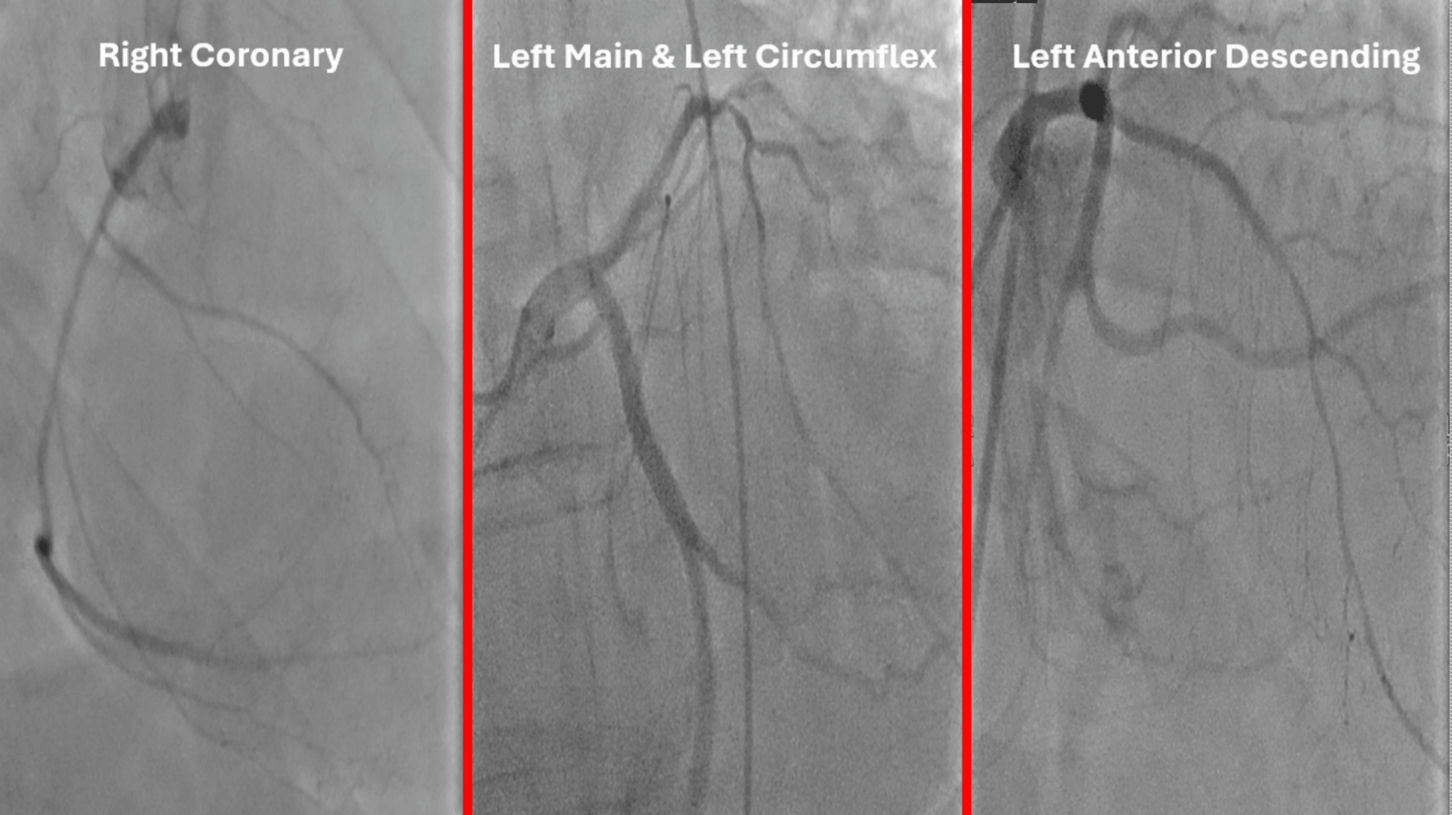
Coronary angiogram reports

An early in-patient invasive coronary angiogram did not demonstrate any obstructive coronary lesion accountable for his cardiac arrest (Figure 4).

Treatment

Given the events, elaborate thought was put into deciding if it would be appropriate for our patient to have a secondary prevention ICD. This needed a holistic discussion with the patient and his family, with input from multiple specialties. Firstly, he was comprehensively discussed in our multidisciplinary meeting involving cardiologists with expertise in complex devices, electrophysiology, intervention, and imaging. The outcome of that meeting was that he qualified for a secondary prevention ICD. However, due to vascular and high infection risk, a subcutaneous implantable cardioverter defibrillator (S-ICD) was deemed to be more appropriate, especially as there were no pacing indications. Subsequently, we sought the opinion of the neurophysiologists to confirm the absence of epileptiform activity during this presentation. From the perspective of his lung function, the respiratory team advised that although he had significant comorbidities, his extensive support mechanisms meant it was highly likely that his life expectancy would be more than a year. Finally, anesthetists were consulted to ensure that our patient could have a suitable anesthetic option for an S-ICD procedure. After a pre-assessment, it was decided the safest option was to proceed with a truncal regional block. Putting information from various specialties together, it was deemed that our patient would benefit from a secondary prevention ICD, and he accepted this offer after some consideration with his family. There were no pacing indications, which allowed us to explore the option of an S-ICD rather than a transvenous ICD, the former being preferable as it reduces the risk of systemic infection, especially with an existing tunneled central venous catheter.

**Figure 5:**
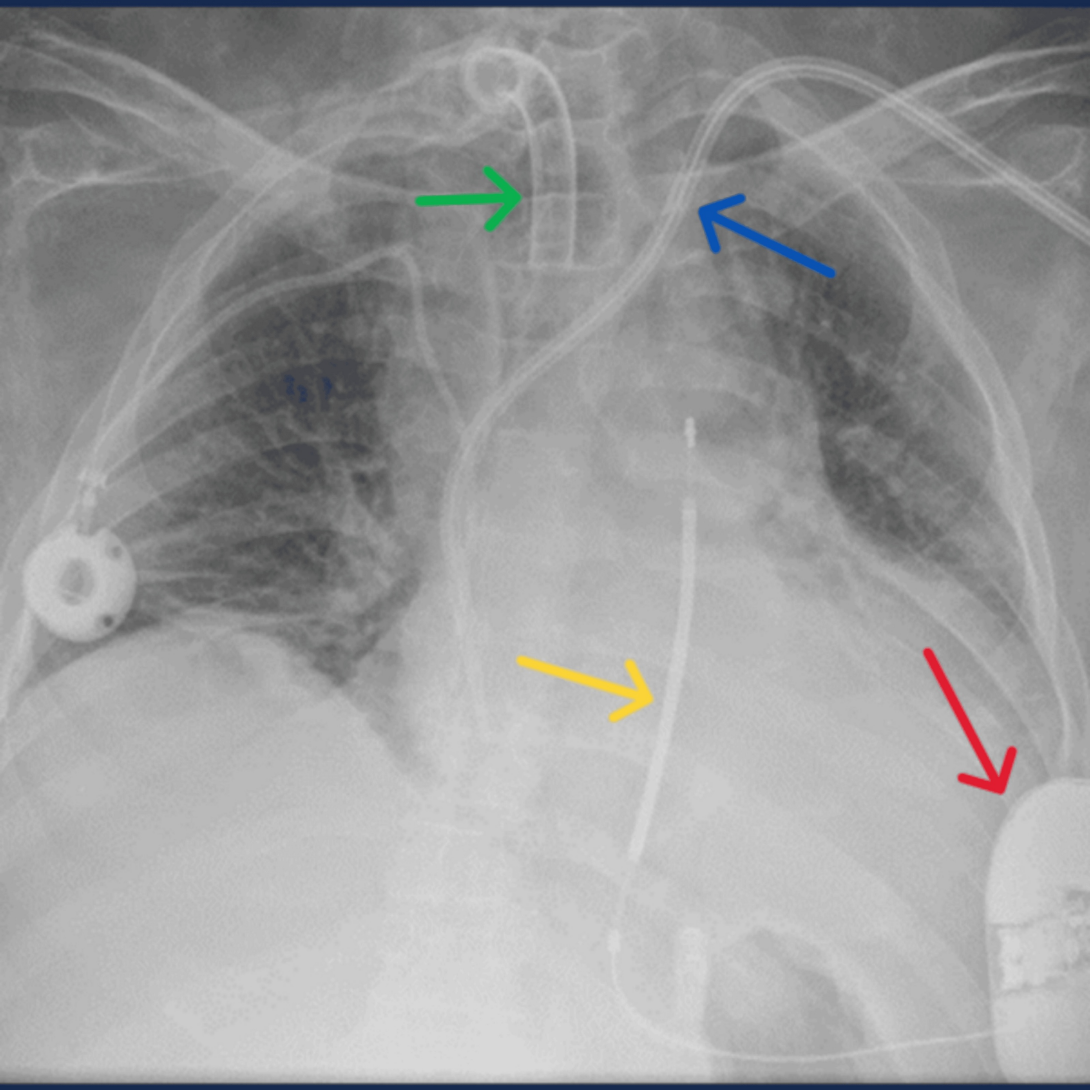
A chest radiograph obtained following the implantation of a subcutaneous implantable cardioverter defibrillator (S-ICD). Red arrow: S-ICD pulse generator box; yellow arrow: S-ICD electrode; blue arrow: left internal jugular tunneled central venous catheter; green arrow: port-a-cath in the right subclavian vein for long-term venous access

He successfully passed all three sensing vectors needed; ultimately, an S-ICD was implanted in the left mid-axillary position between the latissimus dorsi and the serratus anterior muscles. Following the positioning of the generator box in the pocket, an induced ventricular fibrillation was successfully cardioverted via the S-ICD with a single 65J shock. After 15 days as an inpatient, our patient was safely discharged with a planned follow-up in the local device clinic (Figure 5).

Outcome and follow-up

During the first month since discharge, our patient suffered a localized infection over his S-ICD wound site. The wound healed satisfactorily after oral antibiotics with a short course of intravenous antibiotics. Two months following discharge, he sustained a left extracapsular neck of femur fracture from an accident, where he underwent an intramedullary nail fixation. Throughout the following year, he was closely managed by the infectious diseases team with multiple prolonged courses of broad-spectrum intravenous antibiotics for presumed recurrent extra-cardiac infections-possible sources included osteomyelitis, chest, urine, and the gastrointestinal tract. In addition, he was also hospitalized for an episode of acute pancreatitis. Despite these challenges, our patient was able to recover reasonably well and carry out his regular activities with his pre-admission level of independence. 

Unfortunately, 14 months post discharge, our patient deteriorated rapidly with a septic shock secondary to terminal small bowel ischemia. He was discharged home on a fast-track palliative care service from intensive care. He was able to die comfortably in his own home surrounded by his family members. Following implantation, his S-ICD device checks were satisfactory throughout the year, and he did not need any device therapies.

## Discussion

Motor neuron disease is a neurodegenerative disease that can selectively damage the central nervous system, peripheral nervous system, or both [8]. Cardiac involvement in such patients has been controversial; however, it is postulated that cardiac denervation attributing to the involvement of the sympathetic nervous system in the early stages is possible.

Fabry disease, on the other hand, leads to the accumulation of Gb3 in the affected tissues, including the heart. Cardiac involvement usually manifests as left ventricular hypertrophy, myocardial fibrosis, heart failure, and arrhythmias, limiting the quality of life and representing the most common causes of death [1,9]. In adults, the earliest clinical manifestations of cardiac involvement are ECG abnormalities (signs of left ventricular hypertrophy, short PR interval or atrioventricular (AV) conduction abnormalities, and deep T inversions in precordial leads) [10]. As patients get older, progressive interstitial and replacement myocardial fibrosis develops, usually starting in the mid-myocardial layer of the basal posterolateral left ventricular wall [10]. Conduction defects ranging from short PR interval without pre-excitation, chronotropic incompetence, and arrhythmias including AV block, bundle branch block, and atrial fibrillation to ventricular arrhythmias are known to occur [10]. Twenty-four-hour Holter monitoring of patients with FD had shown that non-sustained ventricular tachycardias (NSVT) are not uncommon in this patient cohort; even cases of fatal malignant arrhythmias resistant to ICD therapies have been reported.

A systematic review examining patients with FD who suffered from sudden cardiac deaths suggested that fatal ventricular arrhythmias were as high as 62% of all sudden cardiac deaths [11,12]. Nevertheless, the introduction of automated internal cardiac defibrillators (AICD) has been shown to decrease the incidence of circulatory collapse in FD patients with known life-threatening arrhythmias [10].

It is extremely uncommon to encounter a patient with the co-existence of MND and multisystem FD. The prognosis when a patient has both conditions is unclear. Our case demonstrates that a multidisciplinary team approach paved the way to provide life-preserving treatment in an individual with significant co-morbidities from dual pathology of MND and multisystem FD. A multidisciplinary team approach allowed us to examine the possible causes of his cardiac arrest from a wide perspective. A multidisciplinary team approach also aided the meticulous decision-making required for an ICD implantation in this patient with a complex prognosis. The current European Society of Cardiology guideline for ICD demands that the patient’s prognosis should be more than a year [13]. Working in collaboration, cardiology, neurology, anesthesiology, and respiratory doctors were able to provide consultation and reach a consensus that the patient was likely to live longer than a year.

The cardiology devices team, along with the electrophysiologists, deliberated in-depth before focusing on implanting a subcutaneous ICD. This decision was predominantly influenced by the fact that the patient had already had two tunneled central venous catheters in situ, and this factor itself increased the risk for systemic infection. A systemic infection is highly likely to compromise tunneled lines and implantable cardiac devices, both having catastrophic implications for our patient. Moreover, there was also no pacing indication warranting a transvenous system. Finally, ECGs obtained from the automated defibrillator used in the community at the time of cardiac arrest confirmed the life-threatening arrhythmia to be ventricular fibrillation. This meant that there was no role for anti-tachycardia pacing (ATP) therapy. The procedure was carried out in a punctilious manner, after which our patient made a good recovery and was discharged the following day.

This case showcases that an MDT approach is pivotal in achieving safe and effective management of complex medical cases such as our patient. This is especially relevant in modern medicine, where our aging population is inevitably developing increasingly complex comorbidities.

## Conclusions

Finding a patient who has both multisystem FD and MND is quite rare. When a patient has both illnesses, the prognosis is not certain. To deliver safe and efficient care in the increasingly complicated field of contemporary medicine, a multidisciplinary team approach including a variety of professionals must be used. When feasible, the S-ICD should be taken into consideration because of the typically decreased risk of infection and lead-related problems.
